# Validation of a new objective method to assess lipid layer thickness without the need of an interferometer

**DOI:** 10.1007/s00417-021-05378-8

**Published:** 2021-09-06

**Authors:** José Vicente García-Marqués, Cristian Talens-Estarelles, Santiago García-Lázaro, Alejandro Cerviño

**Affiliations:** Department of Optics and Optometry and Vision Sciences, University of Valencia, C/Dr Moliner, 50, 46100 Burjassot, Valencia Spain

**Keywords:** Dry eye disease, High-speed videokeratoscopy, Image processing, Lipid layer thickness, Meibomian gland dysfunction, Tear film

## Abstract

**Purpose:**

This study aimed to develop and validate new metrics to objectively assess the lipid layer thickness (LLT) through the analysis of grey intensity values obtained from the Placido disk pattern reflected onto the tear film.

**Methods:**

Ocular surface parameters were measured using Oculus Keratograph 5 M in 94 healthy volunteers (43.8 ± 26.8 years). Subjects’ LLT was subjectively classified into 4 groups using an interferometry-based grading scale. New metrics based on the intensity of the Placido disk images were calculated and compared between groups. The repeatability of the new metrics and their diagnostic ability was analysed through receiver operating characteristics (ROC) curves. The level of agreement between the new objective tool and the existing subjective classification scale was analysed by means accuracy, weighted Kappa index and *F*-measure.

**Results:**

Mean pixel intensity, median pixel intensity and relative energy at 5.33 s after blinking achieved the highest performance, with a correlation with LLT between *r* = 0.655 and 0.674 (*p* < 0.001), sensitivity between 0.92 and 0.94, specificity between 0.79 and 0.81, area under the ROC curve between 0.89 and 0.91, accuracy between 0.76 and 0.77, weighted Kappa index of 0.77 and *F*-measure between 0.86 and 0.87.

**Conclusion:**

The analysis of grey intensity values in videokeratography can be used as an objective tool to assess LLT. These new metrics could be included in a battery of clinical tests as an easy, repeatable, objective and accessible method to improve the detection and monitoring of dry eye disease and meibomian gland dysfunction.

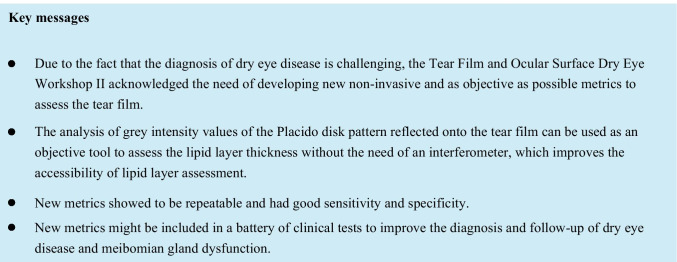

## Introduction

The lipid layer is the outermost layer of the tear film (TF) and is almost entirely derived from meibum, which is secreted by the meibomian glands. The lipid layer plays a vital role in the stabilization of the TF. It also spreads the whole TF over the ocular surface, lowers the surface tension at the air interface of the TF and prevents the aqueous layer from evaporating [1, 2].

Given the key role of the lipid layer in maintaining the properties of the TF, the assessment of the lipid layer thickness (LLT) is essential in dry eye disease (DED) and Meibomian gland dysfunction (MGD) [3, 4]. One of the most common methods for assessing the lipid layer is the evaluation of the colour and brightness of its interference patterns using an interferometer.

The Tearscope Plus™ is an interferometer developed to assess the LLT [4]. However, this is a subjective technique which requires an experienced clinician to classify the interference patterns. It has been reported that subjective diagnostic tests, such as grading scales, rely on the examiner’s ability, which might decrease inter and intra-observer repeatability [3, 5–7]. Likewise, in some cases, the grading of the interference patterns is difficult to perform, especially when dealing with thinner lipid layers [3, 8]. Currently, only the LipiView® system can provide quantitative values of the LLT. However, it has a small area of measurement and it only measures the LLT in blinking conditions [9, 10].

Lately, several studies have tried to solve the aforementioned problems by developing algorithms, based on the analysis of the texture, structure or colour of the interference patterns, which objectively assess the LLT [8, 11–19]. Likewise, other authors have used high-resolution microscopy systems to characterize the LLT [20] or have combined optical coherence tomography with interferometry to develop novel imaging systems [19, 21]. Nonetheless, none of these methods has been globally accepted and most of them are considerably time-consuming. Moreover, they require interferometers to be performed, which are too costly and sophisticated to be implemented in the clinic, being more suitable for research purposes.

During corneal topography measurement, the TF acts as a mirror and reflects the projected Placido disk ring pattern. Placido disk rings show lighter than the background. The healthy TF surface forms a well-structured and reflected pattern with good intensity of reflection, while an altered TF produces an irregular pattern with low reflectivity [22]. Accordingly, the primary aim of the present study was to develop and validate a novel method to objectively assess the lipid layer through the analysis of grey intensity values obtained from the Placido disk pattern reflected onto the TF, without the need of an interferometer, thus making the method widely accessible.

The base of the method is that a thicker lipid layer has more lipids [1], which will reflect the light of the Placido disk ring pattern with higher intensity. We hypothesized that high grey intensity values might be related to a thicker lipid layer, while low grey intensity values might be related to a thinner lipid layer. This method was developed following previous research, which shows that the analysis of grey level intensity values of videokeratoscopy images may significantly improve the diagnosis of DED in comparison to other image analysis approaches [22].

## Material and methods

Ninety-four healthy volunteers ranging in age from 18 to 90 years (43.8 ± 26.8 years) were enrolled in this study. Only the right eye of participants was assessed to avoid subjects’ data duplication. Subjects had no prior history of ocular disease or injury in the last 3 months. No exclusion based on ocular surface parameters was made to evaluate different TF status. Contact lens users were instructed not to wear their contact lenses within a week before the examination. The work was performed in accordance with the tenets of the Declaration of Helsinki and was approved by the Ethics Committee of the University of Valencia. Written consent of each subject was obtained after a verbal explanation of the study protocol.

### Ocular surface measurements

Participants’ ocular surface was evaluated using Oculus Keratograph 5 M (K5 M; Oculus GmbH, Wetzlar, Germany). Measurements were taken by the same experienced researcher following the guidelines of the Tear Film and Ocular Surface Dry Eye Workshop II (TFOS DEWS II) Diagnostic Methodology report [3] and were performed in the following order to avoid TF destabilization: Ocular Surface Disease Index (OSDI), Dry Eye Questionnaire-5 (DEQ-5), total bulbar redness, tear meniscus height (TMH), LLT, non-invasive keratograph break-up time (NIKBUT), meibomian glands expressibility and upper eyelid meibography. The illuminance, temperature and humidity of the room were maintained constant at 200 lx, 24.1 ± 1.6 °C and 44.9 ± 5.0%, respectively.

OSDI and DEQ-5 were used for scoring the ocular surface symptoms of subjects. Bulbar redness was assessed three consecutive times, and an average value was calculated [23], while TMH was obtained by capturing the meniscus immediately post-blink [24].

The LLT was recorded using Oculus Keratograph 5 M and assessed through the lipid layer interference pattern, which was subjectively classified by a masked and experienced examiner into 4 groups using a standardised grading scale [6, 25]: 1 = open meshwork (13–15 nm); 2 = closed meshwork (30–50 nm); 3 = wave (50–80 nm); and 4 = colour fringe (90–140 nm).

The moment of the first break-up of the TF (first NIKBUT) and the average time of all break-ups (mean NIKBUT) were also obtained. A total of three measurements were carried out, one every 3 min so that the TF stabilized between assessments, and the mean and median values of these three measurements were calculated [3].

The expressibility of the central 8 meibomian glands of the upper eyelid was assessed using a subjective grading scale [6, 26, 27]. Upper eyelid meibography was captured using non-contact infrared meibography, and meibomian glands drop-out was objectively calculated using ImageJ tool (Wayne Rasband, National Institutes of Health, Bethesda, MD) as the ratio between gland loss area and eyelid area [28].

### Data analysis using the proposed algorithm

Oculus Keratograph 5 M was used to record a video of the NIKBUT measurement at 32 frames per second with a spatial resolution of 680 × 512 pixels. This video was recorded and saved to be later analysed. The proposed software was developed using Matlab R2019a® (MathWorks, Natick, MA). The software automatically decomposed the video into frames with a time interval of 0.031 s between them. The examiner manually selected the frames at 0.33, 5.33, 10.33, 15.33 and 20.33 s after blinking. The frame of 0.33 was selected since the eye was completely open after this time in all videos. Likewise, intervals of 5.00 s from this moment on were chosen to analyse whether the grey intensity values changed over time.

Once the frames of interest were selected, the software automatically processed the images. First, RGB images were transformed into grey-level images. Given that input images contained irrelevant information of external areas, the centre of the Placido disk ring pattern was isolated by the examiner through Matlab. After clicking the centre of the image, the software automatically selected a square of 241 × 241 pixels surrounding the centre of the rings (region of interest, ROI), as the area to perform the image processing.

Next, a band-pass filter was used to eliminate the background illumination and highlight the rings. Furthermore, the images were then smoothed by applying a 4-pixel sigma Gaussian filter to remove the remaining noise from the background [29]. After that, the final ROI was selected by the examiner, who manually selected the region of the image comprising solely the pupil, to avoid the influence of the iris on the results.

Finally, to increase the differences between normal and altered TFs, each pixel value of the resulting image was multiplied by 255 and divided by 85, thus enhancing the contrast between rings and non-ring spaces. These values were selected since they produced the highest possible contrast enhancement.

**Fig. 1:**
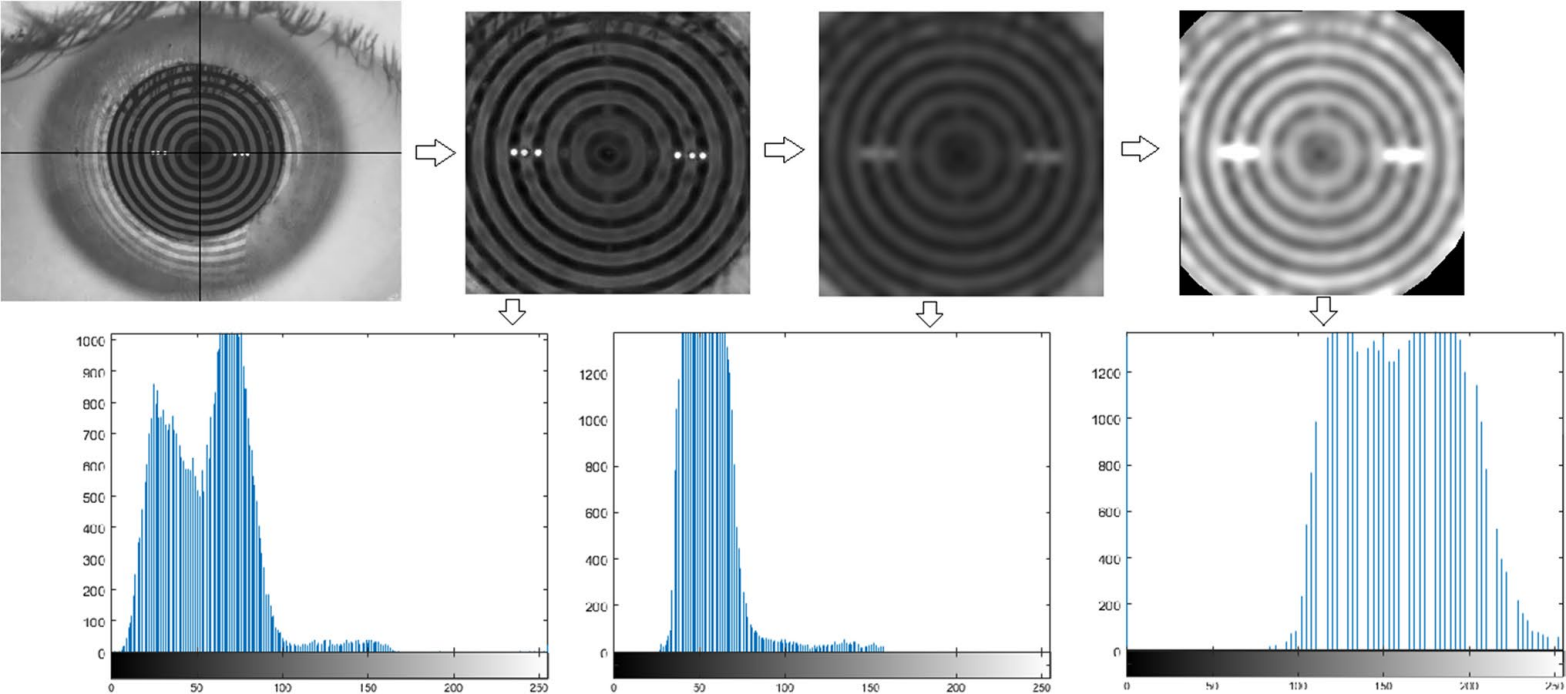
Images and histograms of the main steps of the image processing in a random frame. From left to right: selection of the centre of the image; band-pass filter implementation; Gaussian filter implementation; selection of the final ROI and contrast enhancement (final image). In the histograms, axis “*x*” represents the grey level intensities (0–255), while axis “*y*” shows the number of pixels

**Fig. 2:**
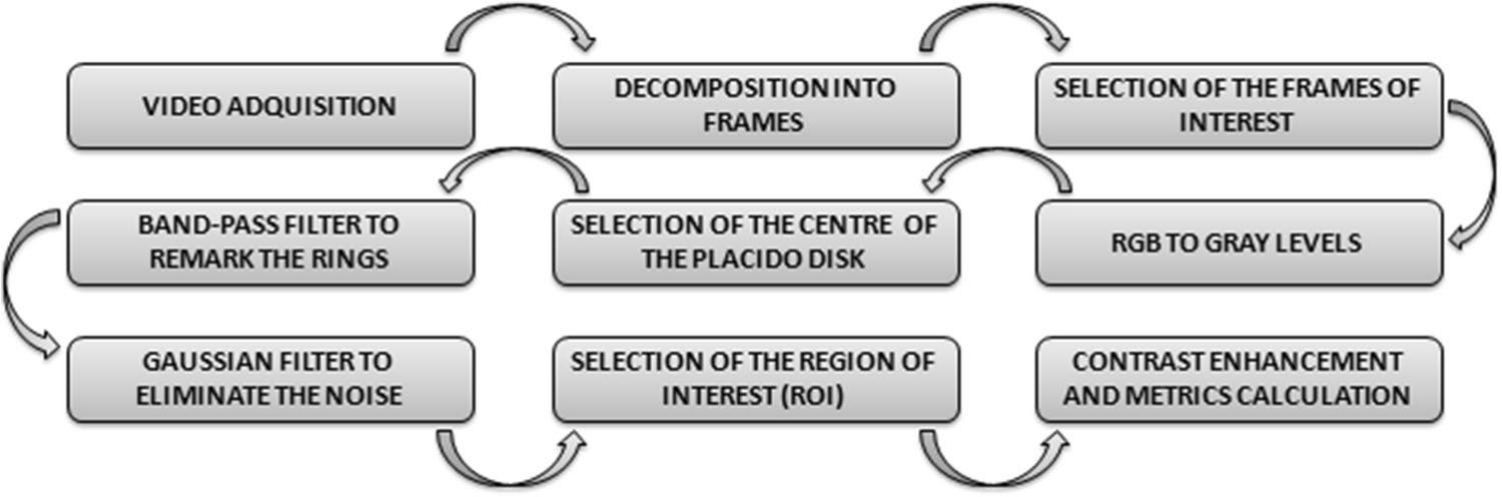
The main steps of the image processing

Once the images were processed, histograms were obtained from their pixel intensity values and metrics were calculated (Fig. 1). Figure 2 shows a summary of the main steps of image processing.

The base of our method is that a thicker lipid layer has more lipids [1], which will reflect the Placido disk rings with higher intensity. Thus, higher grey intensity values might be related to a thicker LLT, while lower grey intensity values could be related to a thinner LLT.

Mean, standard deviation (SD), median, mode, kurtosis and skewness of the histogram of the grey level intensity values were calculated. The minimum grey level in the image was also calculated. Besides, energy, relative energy, entropy and SD irregularity were calculated as follows [22]:

$$\mathrm{Energy as}:\frac{\sum p^2}{n}$$

$$\mathrm{Relative energy as}:\frac{\sum (\frac{p}{\mathrm{pmax}})^2}{n}$$

$$\mathrm{Entropy}:\frac{-\sum p.\mathrm{logr}(p)}{n}$$

$$\mathrm{SD irregularity as}:\frac{\sum \left(\frac{p-x}{\mathrm{pmax}}\right)^2}{n}.$$

where *p* = pixel grey value; *n* = number of pixels of the ROI; pmax = maximum pixel intensity; and *x* = mean pixel intensity values.

**Fig. 3:**
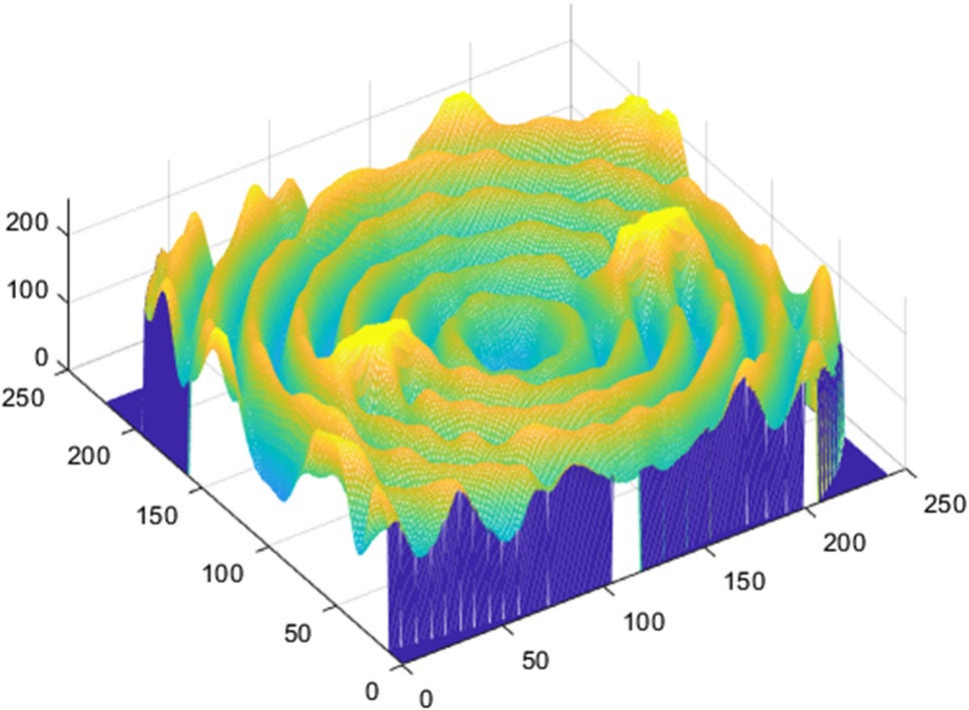
Three-dimensional graphic of the grey intensity values in the image. The “*x*” and “*y*” axes represent the size of the image and “*z*” the grey intensity value for each pixel

Metrics were divided by the number of pixels of the ROI (*n*) so that all images were comparable independently of the size of the ROI. Finally, the total area under the pixel intensity three-dimensional curve of the image was calculated and divided by the number of pixels in the ROI (Fig. 3).

### Statistical analysis

Statistical analysis was carried out using SPSS v26.0 for Windows (IBM Corp, Armonk, NY, USA). Outcomes were shown as the mean ± SD.

#### Differences in new metrics depending on time after blinking

Repeated mixed model ANOVA was used to evaluate the differences in pixel intensity values depending on the moment after blinking. Bonferroni was used to assess the post hoc differences between paired moments.

#### Repeatability of new metrics

As three NIKBUT videos were recorded, the three videos were analysed so as to calculate the repeatability of the software in the calculation Placido disk’ reflectivity metrics. Repeatability of each Placido disk’ reflectivity metric was assessed by calculating the within-subject SD (*S*_w_), coefficient of variation (CoV) and the repeatability coefficient (CoR) [30–32].

#### Correlations of new metrics with DED signs and symptoms

Rho Spearman correlations were used to analyse the correlations between ocular surface signs and symptoms and new metrics, for the whole sample. Moreover, the sample was divided into different groups according to the cut-off values reported by the Diagnostic Methodology report of TFOS DEWS [3].

Differences in Placido disk’ reflectivity metrics between groups was assessed by means of Mann–Whitney *U* test or Kruskal–Wallis test. A *p*-value less than 0.05 was defined as statistically significant.

#### Multiple linear regressions

Multiple linear regressions were performed to assess the predictability of tear film-dynamic metrics to ocular signs that had statistically significant correlations. Multiple linear models were constructed with new metrics as dependent variables and current metrics as independent variables to assess the relative importance of each independent variable and their contribution to the change of dependent variables. The following assumptions were checked: the linear relationship between the independent and dependent variables, normal distribution of residuals, homoscedasticity of residuals and predicted values and absence of multicollinearity between independent variables.

#### Diagnostic ability and validation of new metrics

Each new metric was validated by means receiver operating characteristics (ROC) curves. The probability density functions for an altered (LLT = 1) or normal (LLT ≥ 2) LLT were calculated [3], and different parameters were obtained for each ROC curve: sensitivity, specificity, area under the ROC curve, the cut-off value that optimizes the diagnosis, Youden index and discriminant power [33].

Finally, each Placido disk image was objectively classified into LLT groups depending on the cut-off values obtained in the ROC curves. The level of agreement between this objective classification and the subjective ones was analysed by calculating the accuracy, Kappa index, weighted Kappa index with quadratic weights and *F*-measure for each metric as in previous studies [34–37]. The three indexes denote high level of agreement between tests when the values are near 1 [34–37].

## Results

The described algorithm was applied to ninety-four eyes from 94 volunteers, 54 females (57.4%) and 40 males (42.6%). The mean age was 43.8 ± 26.8 years, ranging from 18 to 90 years. The algorithm was able to obtain objective metrics in all subjects.

### Placido disk reflectivity metrics over time

Table 1 shows the mean values and SD for each Placido disk reflectivity metric at 0.33, 5.33, 10.33, 15.33 and 20.33 s after blinking. Repeated mixed model ANOVA showed statistical higher pixel intensity values at 10.33, 15.33 and 20.33 s than at 0.33 s. Nevertheless, CoV revealed a low variability of metrics over time. Thus, pixel intensity of the Placido disk was stable in the same subject throughout the measuring period. CoV between seconds after blinking achieved values between 4.42 and 16.92%. Total area under pixel intensity curve, mean pixel intensity, SD of pixel intensity, median pixel intensity and skewness had a CoV < 10%, which evidenced that metrics did not change after blinking.

**Table 1:** Mean values for each Placido disk reflectivity metric

**Metric**		**Number of subjects**	**Mean ± SD**	**Significance level**	**Statistically significant post hoc differences** **(*p*-value)**	**CoV between seconds after blinking (%)**
Total area	0.33 s	94	113.63 ± 9.57	< 0.001^1^	1–4 < 0.001	4.42
	5.33 s	90	116.79 ± 8.48		1–5 < 0.001	
	10.33 s	66	117.15 ± 9.18			
	15.33 s	49	119.22 ± 8.30			
	20.33 s	37	120.30 ± 7.87			
Minimum pixel intensity	0.33 s	94	67.12 ± 17.37	0.082^1^		13.58
	5.33 s	90	69.10 ± 16.20			
	10.33 s	66	70.00 ± 18.84			
	15.33 s	49	75.12 ± 18.98			
	20.33 s	37	70.58 ± 18.16			
Energy	0.33 s	94	240.78 ± 8.89	< 0.001^1^	1–3 < 0.001	14.84
	5.33 s	90	244.72 ± 7.06		1–4 < 0.001	
	10.33 s	66	249.45 ± 5.02		1–5 < 0.001	
	15.33 s	49	251.82 ± 3.32			
	20.33 s	37	253.82 ± 2.02			
Relative energy	0.33 s	94	0.46 ± 0.26	< 0.001^1^	1–3 < 0.001	15.78
	5.33 s	90	0.54 ± 0.26		1–4 < 0.001	
	10.33 s	66	0.57 ± 0.28		1–5 < 0.001	
	15.33 s	49	0.63 ± 0.27			
	20.33 s	37	0.68 ± 0.27			
Entropy	0.33 s	94	2.0 × 10^−4^ ± 1.2 × 10^−4^	0.906^1^		16.92
	5.33 s	90	2.0 × 10^−4^ ± 1.2 × 10^−4^			
	10.33 s	66	2.0 × 10^−4^ ± 1.2 × 10^−4^			
	15.33 s	49	2.0 × 10^−4^ ± 1.2 × 10^−4^			
	20.33 s	37	2.0 × 10^−4^ ± 1.2 × 10^−4^			
SD irregularity	0.33 s	94	0.08 ± 0.12	0.002^1^	1–4 = 0.005	15.26
	5.33 s	90	0.10 ± 0.12		1–5 = 0.001	
	10.33 s	66	0.12 ± 0.14			
	15.33 s	49	0.13 ± 0.12			
	20.33 s	37	0.17 ± 0.17			
Mean pixel intensity	0.33 s	94	130.38 ± 26.74	< 0.001^1^	1–3 < 0.001	9.31
	5.33 s	90	137.24 ± 26.96		1–4 < 0.001	
	10.33 s	66	140.58 ± 29.76		1–5 < 0.001	
	15.33 s	49	146.55 ± 29.27			
	20.33 s	37	153.06 ± 31.80			
SD of pixel intensity	0.33 s	94	26.89 ± 4.63	< 0.001^1^	1–3 < 0.001	8.73
	5.33 s	90	29.62 ± 4.84		1–4 < 0.001	
	10.33 s	66	30.43 ± 4.50		1–5 < 0.001	
	15.33 s	49	31.19 ± 4.92			
	20.33 s	37	32.62 ± 4.83			
Median pixel intensity	0.33 s	94	127.12 ± 27.47	< 0.001^1^	1–4 < 0.001	9.73
	5.33 s	90	136.27 ± 27.97		1–5 < 0.001	
	10.33 s	66	139.68 ± 31.12			
	15.33 s	49	145.59 ± 30.56			
	20.33 s	37	152.52 ± 33.03			
Mode pixel intensity	0.33 s	94	142.40 ± 40.12	< 0.001^1^	1–3 = 0.004	16.79
	5.33 s	90	150.03 ± 47.08		1–4 = 0.001	
	10.33 s	66	152.90 ± 50.89		1–5 < 0.001	
	15.33 s	49	164.27 ± 55.38			
	20.33 s	37	175.22 ± 60.98			
Kurtosis	0.33 s	94	0.017 ± 0.001	< 0.001^1^	1–4 < 0.001	11.37
	5.33 s	90	0.015 ± 0.003		1–5 < 0.001	
	10.33 s	66	0.015 ± 0.003			
	15.33 s	49	0.014 ± 0.003			
	20.33 s	37	0.014 ± 0.003			
Skewness	0.33 s	94	0.14 ± 0.02	< 0.001^1^	1–4 < 0.001	6.90
	5.33 s	90	0.13 ± 0.01		1–5 < 0.001	
	10.33 s	66	0.13 ± 0.02			
	15.33 s	49	0.13 ± 0.02			
	20.33 s	37	0.12 ± 0.02			

*CoV* coefficient of variation, *s* seconds, *SD* standard deviation.

^1^Repeated mixed model ANOVA.

### Repeatability of Placido disk reflectivity metrics

Table 2 shows the repeatability scores for each metric. All metrics showed acceptable repeatability since *S*_w_, CoR and CoV values were low, and the variability between the three measurements was not high. *S*_w_ achieved values between 2 × 10^−6^ and 7.07, CoR between 6 × 10^−6^ and 19.59 and CoV between 0.09 and 5.15.

**Table 2:** Repeatability of each Placido disk reflectivity metric

Metric	*S*_w_	CoR	CoV (%)
**At 0.33 s**			
Total area	0.63	1.74	0.54
Minimum pixel intensity	2.95	8.16	4.42
Energy	0.23	0.64	0.09
Relative energy	0.01	0.04	2.85
Entropy	2 × 10^−6^	6 × 10^−6^	1.95
SD irregularity	0.003	0.01	3.66
Mean pixel intensity	1.28	3.55	0.97
SD pixel intensity	0.34	0.96	1.25
Median pixel intensity	1.30	3.59	0.99
Mode pixel intensity	7.02	19.44	5.11
Kurtosis	3 × 10^−4^	9 × 10^−4^	2.39
Skewness	0.002	0.006	1.56
**At 5.33 s**			
Total area	0.54	1.49	0.47
Minimum pixel intensity	2.82	7.82	4.23
Energy	0.23	0.64	0.09
Relative energy	0.01	0.04	2.84
Entropy	2 × 10^−6^	6 × 10^−6^	1.94
SD irregularity	0.003	0.01	3.64
Mean pixel intensity	1.06	2.93	0.80
SD pixel intensity	0.34	0.93	1.21
Median pixel intensity	1.29	3.56	0.98
Mode pixel intensity	7.01	19.40	5.10
Kurtosis	3 × 10^−4^	8 × 10^−4^	2.01
Skewness	0.001	0.004	1.11
**At 10.33 s**			
Total area	0.59	1.64	0.51
Minimum pixel intensity	2.76	7.64	4.15
Energy	0.23	0.64	0.09
Relative energy	0.01	0.04	2.89
Entropy	2 × 10^−6^	8 × 10^−6^	2.49
SD irregularity	0.003	0.01	3.97
Mean pixel intensity	1.33	3.67	1.00
SD pixel intensity	0.35	0.97	1.26
Median pixel intensity	1.30	3.61	0.99
Mode pixel intensity	7.03	19.48	5.12
Kurtosis	3 × 10^−4^	9 × 10^−4^	2.09
Skewness	0.002	0.004	1.21
**At 15.33 s**			
Total area	0.60	1.67	0.52
Minimum pixel intensity	2.81	7.78	4.19
Energy	0.23	0.64	0.09
Relative energy	0.01	0.04	2.90
Entropy	3 × 10^−6^	8 × 10^−6^	2.56
SD irregularity	0.003	0.01	4.05
Mean pixel intensity	1.35	3.75	1.02
SD pixel intensity	0.42	1.16	1.50
Median pixel intensity	1.32	3.66	1.01
Mode pixel intensity	7.06	19.57	5.14
Kurtosis	3 × 10^−4^	9 × 10^−4^	2.07
Skewness	0.002	0.005	1.42
**At 20.33 s**			
Total area	0.68	1.87	0.58
Minimum pixel intensity	3.14	8.69	4.69
Energy	0.23	0.64	0.09
Relative energy	0.01	0.04	2.93
Entropy	3 × 10^−6^	8 × 10^−6^	2.62
SD irregularity	0.003	0.01	4.21
Mean pixel intensity	1.44	4.00	1.09
SD pixel intensity	0.42	1.16	1.51
Median pixel intensity	1.34	3.71	1.02
Mode pixel intensity	7.07	19.59	5.15
Kurtosis	3 × 10^−4^	9 × 10^−4^	2.37
Skewness	0.004	0.01	2.73

*CoR* repeatability coefficient, *CoV* coefficient of variation, *s* seconds, *SD* standard deviation; *S*_*w*_ within-subject standard deviation

### Correlations between new metrics and DED signs and symptoms

Following the results of the previous sections, showing no variation of the metrics over time, only the metrics at 0.33, 5.33 and 10.33 s after blinking were further assessed. Metrics at 15.33 and 20.33 s were excluded from further analysis as most patients need to suppress blinking forcefully, and thus, they do not represent in most cases a real scenario.

Spearman’s significant correlations between each Placido disk reflectivity metric and DED signs and symptoms are shown in Table 3. Generally, there were moderate negative correlations between new metrics based on the grey intensity of pixels of Placido disk images and age, meibomian glands drop-out percentage, bulbar redness, TMH and OSDI. Meanwhile, Placido disk reflectivity metrics were positively correlated with LLT and NIKBUT. The correlation with LLT was the strongest. Given that LLT was statistically correlated with age (*r* =  − 0.298, p = 0.002), glands drop-out (*r* =  − 0.271, *p* = 0.004), mean first NIKBUT (*r* =  − 0.209, *p* = 0.008), median first NIKBUT, mean mean NIKBUT and median mean NIKBUT, it might be possible that the correlation of new metrics with the other ocular surface metrics was as consequence of the correlation with LLT. Nevertheless, LLT was not statistically correlated with bulbar redness, TMH and OSDI.

**Table 3:** Statistically significant Rho Spearman correlations between Placido disk reflectivity metrics and DED signs and symptoms

New metrics	Current metrics	Correlation coefficient (*r*)	Significance level
**At 0.33 s**			
Total area	Age	− 0.372	< 0.001
	Drop-out percentage	− 0.277	0.007
	LLT	0.413	< 0.001
	Mean mean NIKBUT	0.209	0.048
Minimum pixel intensity	Age	− 0.387	< 0.001
	Drop-out percentage	− 0.236	0.022
	LLT	0.345	0.001
Energy	Age	− 0.346	< 0.001
	Drop-out percentage	− 0.236	< 0.001
	LLT	0.408	< 0.001
Relative energy	Age	− 0.356	< 0.001
	Drop-out percentage	− 0.266	0.010
	LLT	0.407	< 0.001
	TMH	− 0.205	0.048
Entropy	Age	− 0.670	< 0.001
	Drop-out percentage	− 0.517	< 0.001
	Bulbar redness	− 0.615	< 0.001
	TMH	− 0.395	< 0.001
	Mean first NIKBUT	0.223	0.033
	Median first NIKBUT	0.226	0.030
	OSDI	− 0.316	0.002
SD irregularity	Age	− 0.448	< 0.001
	Drop-out percentage	− 0.322	0.002
	Bulbar redness	− 0.306	0.003
	LLT	0.454	< 0.001
Mean pixel intensity	Age	− 0.388	< 0.001
	Drop-out percentage	− 0.285	0.005
	Bulbar redness	− 0.210	0.043
	LLT	0.426	< 0.001
	Mean mean NIKBUT	0.210	0.046
SD pixel intensity	Age	− 0.507	< 0.001
	Drop-out percentage	− 0.331	0.001
	Bulbar redness	− 0.400	< 0.001
	LLT	0.446	< 0.001
Median pixel intensity	Age	− 0.383	< 0.001
	Drop-out percentage	− 0.294	0.004
	LLT	0.426	< 0.001
Mode pixel intensity	Age	− 0.305	0.003
	Drop-out percentage	− 0.234	0.023
	LLT	0.418	< 0.001
Kurtosis	LLT	− 0.515	< 0.001
	Mean mean NIKBUT	− 0.252	0.016
	Median mean NIKBUT	− 0.251	0.017
Skewness	LLT	− 0.510	< 0.001
	Mean mean NIKBUT	− 0.237	0.024
	Median mean NIKBUT	− 0.230	0.029
**At 5.33 s**			
Total area	LLT	0.647	< 0.001
	Mean first NIKBUT	0.265	0.011
	Mean mean NIKBUT	0.233	0.029
Minimum pixel intensity	LLT	0.589	< 0.001
	Mean first NIKBUT	0.229	0.030
Energy	LLT	0.548	< 0.001
	Mean first NIKBUT	0.223	0.019
Relative energy	LLT	0.655	< 0.001
	Mean first NIKBUT	0.237	0.024
Entropy	Age	− 0.642	< 0.001
	Drop-out percentage	− 0.572	< 0.001
	Bulbar redness	− 0.564	< 0.001
	TMH	− 0.403	< 0.001
	Mean first NIKBUT	0.221	0.036
	Median first NIKBUT	0.219	0.038
	OSDI	− 0.260	0.013
SD irregularity	Age	− 0.327	0.002
	Drop-out percentage	− 0.249	0.018
	LLT	0.662	< 0.001
Mean pixel intensity	LLT	0.665	< 0.001
	Mean first NIKBUT	0.235	0.026
SD of pixel intensity	Age	− 0.408	< 0.001
	Drop-out percentage	− 0.272	0.009
	Bulbar redness	− 0.247	0.019
	LLT	0.572	< 0.001
Median pixel intensity	LLT	0.674	< 0.001
	Mean first NIKBUT	0.246	0.020
	Mean mean NIKBUT	0.220	0.040
Mode pixel intensity	LLT	0.657	< 0.001
	Mean first NIKBUT	0.233	0.027
Kurtosis	LLT	− 0.672	< 0.001
Skewness	LLT	− 0.673	< 0.001
**At 10.33 s**			
Total area	LLT	0.645	< 0.001
Minimum pixel intensity	LLT	0.523	< 0.001
Energy	LLT	0.660	< 0.001
Relative energy	LLT	0.654	< 0.001
Entropy	Age	− 0.668	< 0.001
	Drop-out percentage	− 0.596	< 0.001
	Bulbar redness	− 0.542	< 0.001
	TMH	− 0.536	< 0.001
	Mean first NIKBUT	0.260	0.034
	Median first NIKBUT	0.282	0.021
	OSDI	− 0.300	0.014
SD irregularity	Age	− 0.282	0.021
	LLT	0.689	< 0.001
Mean pixel intensity	LLT	0.684	< 0.001
SD of pixel intensity	Age	− 0.371	0.002
	LLT	0.644	< 0.001
Median pixel intensity	LLT	0.687	< 0.001
Mode pixel intensity	LLT	0.654	< 0.001
Kurtosis	LLT	− 0.659	< 0.001
Skewness	LLT	− 0.665	< 0.001

*LLT* lipid layer thickness, *NIKBUT* non-invasive keratograph break-up time, *OSDI* ocular surface disease index, *s* seconds, *TMH* tear meniscus height

Entropy was the only metric which was not correlated with LLT. Likewise, new metrics were not correlated with meibomian glands expressibility or DEQ-5 score. The metrics measured at 5.33 and 10.33 can be considered the best to describe the LLT since they revealed the strongest correlations.

### Differences between groups

The new metrics were analysed according to age and the different ocular surface parameters. Table 4 shows the statistically significant differences in Placido disk reflectivity metrics between classification groups. These outcomes were in accordance with correlations. Statistically higher pixel intensity values were found in young subjects, lower glands drop-out, high NIKBUT, low TMH and thick LLT. However, no statistical differences were found between grade 3 (wave) and 4 (colour fringe) interference patterns in the assessment of LLT (*p* > 0.005).

**Table 4:** Statistically significant differences in Placido disk reflectivity metrics for each ocular surface parameter

New metrics	*n*	Groups	Mean ± SD	Significance level	Statistically significant post hoc differences (*p*-value)
**Age**					
**At 0.33 s**					
Total area	60	< 49 years	117.43 ± 11.62	< 0.001^1^	
	34	> 49 years	111.47 ± 7.46		
Minimum pixel intensity	60	< 49 years	75.44 ± 20.64	< 0.001^1^	
	34	> 49 years	62.40 ± 13.20		
Energy	60	< 49 years	254.18 ± 4.38	< 0.001^1^	
	34	> 49 years	245.28 ± 4.38		
Relative energy	60	< 49 years	0.59 ± 0.30	< 0.001^1^	
	34	> 49 years	0.38 ± 0.19		
Entropy	60	< 49 years	3.0 × 10^−4^ ± 1.5 × 10^−4^	< 0.001^1^	
	34	> 49 years	1.0 × 10^−4^ ± 4 × 10^−5^		
SD irregularity	60	< 49 years	0.13 ± 0.16	< 0.001^1^	
	34	> 49 years	0.04 ± 0.03		
Mean pixel intensity	60	< 49 years	143.32 ± 33.52	< 0.001^1^	
	34	> 49 years	119.92 ± 17.24		
SD of pixel intensity	60	< 49 years	30.05 ± 5.21	< 0.001^1^	
	34	> 49 years	25.10 ± 3.09		
Median pixel intensity	60	< 49 years	142.32 ± 34.97	< 0.001^1^	
	34	> 49 years	118.50 ± 17.22		
Mode pixel intensity	60	< 49 years	155.03 ± 53.85	0.006^1^	
	34	> 49 years	125.85 ± 24.52		
**At 5.33 s**					
Entropy	59	< 49 years	3.0 × 10^−4^ ± 2.1 × 10^−4^	< 0.001^1^	
	31	> 49 years	1.0 × 10^−4^ ± 5 × 10^−5^		
SD irregularity	59	< 49 years	0.15 ± 0.15	0.002^1^	
	31	> 49 years	0.07 ± 0.08		
Mean pixel intensity	59	< 49 years	147.34 ± 32.78	0.044^1^	
	31	> 49 years	131.92 ± 21.83		
SD of pixel intensity	59	< 49 years	32.53 ± 5.47	< 0.001^1^	
	31	> 49 years	28.09 ± 3.68		
Mode	59	< 49 years	169.26 ± 58.13	0.043^1^	
	31	> 49 years	139.93 ± 36.77		
**At 10.33 s**					
Entropy	43	< 49 years	3.0 × 10^−4^ ± 1.7 × 10^−4^	< 0.001^1^	
	23	> 49 years	1.0 × 10^−4^ ± 4 × 10^−5^		
SD irregularity	43	< 49 years	0.17 ± 0.19	0.011^1^	
	23	> 49 years	0.08 ± 0.09		
SD of pixel intensity	43	< 49 years	32.62 ± 4.10	0.002^1^	
	23	> 49 years	29.10 ± 4.37		
**Bulbar redness**					
**At 0.33 s**					
Total area	60	< 1	116.59 ± 11.70	0.008^1^	
	34	> 1	111.18 ± 7.73		
Minimum pixel intensity	60	< 1	73.32 ± 20.77	0.004^1^	
	34	> 1	63.60 ± 14.12		
Energy	60	< 1	254.08 ± 4.40	< 0.001^1^	
	34	> 1	245.44 ± 21.94		
Relative energy	60	< 1	0.57 ± 0.29	0.002^1^	
	34	> 1	0.39 ± 0.21		
Entropy	60	< 1	3.0 × 10^−4^ ± 1.5 × 10^−4^	< 0.001^1^	
	34	> 1	1.0 × 10^−4^ ± 4 × 10^−5^		
SD irregularity	60	< 1	0.13 ± 0.16	< 0.001^1^	
	34	> 1	0.05 ± 0.04		
Mean pixel intensity	60	< 1	140.99 ± 33.54	0.001^1^	
	34	> 1	121.23 ± 18.82		
SD of pixel intensity	60	< 1	29.98 ± 5.08	< 0.001^1^	
	34	> 1	25.14 ± 3.27		
Median pixel intensity	60	< 1	139.94 ± 34.98	0.001^1^	
	34	> 1	119.85 ± 18.87		
Mode pixel intensity	60	< 1	152.92 ± 54.38	0.011^1^	
	34	> 1	127.05 ± 25.23		
Skewness	60	< 1	0.14 ± 0.03	0.044^1^	
	34	> 1	0.15 ± 0.01		
**At 5.33 s**					
Entropy	58	< 1	3.0 × 10^−4^ ± 2.1 × 10^−4^	< 0.001^1^	
	32	> 1	1.0 × 10^−4^ ± 5 × 10^−5^		
SD irregularity	58	< 1	0.14 ± 0.15	0.028^1^	
	32	> 1	0.07 ± 0.08		
SD of pixel intensity	58	< 1	31.99 ± 5.58	0.002^1^	
	32	> 1	28.30 ± 3.84		
Entropy	40	< 1	3.0 × 10^−4^ ± 1.7 × 10^−4^	< 0.001^1^	
	25	> 1	1.0 × 10^−4^ ± 4 × 10^−5^		
SD of pixel intensity	40	< 1	131.72 ± 4.24	0.024^1^	
	25	> 1	129.42 ± 5.59		
**Meibomian glands drop-out**					
**At 0.33 s**					
Total area	50	< 1/3	115.94 ± 10.62	0.008^1^	
	44	> 1/3	111.59 ± 8.12		
Minimum pixel intensity	50	< 1/3	71.80 ± 19.69	0.008^1^	
	44	> 1/3	63.00 ± 13.97		
Energy	50	< 1/3	254.12 ± 4.20	0.002^1^	
	44	> 1/3	245.94 ± 4.18		
Relative energy	50	< 1/3	0.54 ± 0.28	0.007^1^	
	44	> 1/3	0.39 ± 0.22		
Entropy	50	< 1/3	2.0 × 10^−4^ ± 1.4 × 10^−4^	< 0.001^1^	
	44	> 1/3	2.0 × 10^−4^ ± 7.0 × 10^−5^		
SD irregularity	50	< 1/3	0.12 ± 0.15	0.001^1^	
	44	> 1/3	0.05 ± 0.04		
Mean pixel intensity	50	< 1/3	136.92 ± 31.60	0.005^1^	
	44	> 1/3	120.87 ± 18.90		
SD of pixel intensity	50	< 1/3	28.65 ± 5.29	0.002^1^	
	44	> 1/3	25.35 ± 3.30		
Median pixel intensity	50	< 1/3	135.95 ± 32.77	0.005^1^	
	44	> 1/3	119.34 ± 18.90		
Mode pixel intensity	50	< 1/3	148.50 ± 48.31	0.020^1^	
	44	> 1/3	125.76 ± 27.54		
**At 5.33 s**					
Entropy	48	< 1/3	3.0 × 10^−4^ ± 2.0 × 10^−4^	< 0.001^1^	
	42	> 1/3	1.0 × 10^−4^ ± 8.0 × 10^−5^		
SD irregularity	48	< 1/3	0.13 ± 0.14	0.031^1^	
	42	> 1/3	0.07 ± 0.09		
SD of pixel intensity	48	< 1/3	31.08 ± 5.39	0.013^1^	
	42	> 1/3	28.34 ± 3.93		
**At 10.33 s**					
Entropy	33	< 1/3	3.0 × 10^−4^ ± 1.6 × 10^−4^	< 0.001^1^	
	33	> 1/3	1.0 × 10^−4^ ± 4.0 × 10^−5^		
SD irregularity	33	< 1/3	0.14 ± 0.16	0.046^1^	
	33	> 1/3	0.09 ± 0.10		
SD of pixel intensity	33	< 1/3	31.48 ± 4.06	0.021^1^	
	33	> 1/3	29.17 ± 4.80		
**TMH**					
**At 0.33 s**					
Entropy	60	> 0.20 mm	3.0 × 10^−4^ ± 1.6 × 10^−4^	< 0.001^1^	
	34	< 0.20 mm	2.0 × 10^−4^ ± 5.0 × 10^−5^		
**At 5.33 s**					
Entropy	55	> 0.20 mm	3.0 × 10^−4^ ± 2.2 × 10^−4^	< 0.001^1^	
	35	< 0.20 mm	1.0 × 10^−4^ ± 5.0 × 10^−5^		
**At 10.33 s**					
Entropy	38	> 0.20 mm	3.0 × 10^−4^ ± 1.7 × 10^−4^	< 0.001^1^	
	28	< 0.20 mm	1.0 × 10^−4^ ± 4.0 × 10^−5^		
**Mean first NIKBUT**					
**At 0.33 s**					
Entropy	41	> 10 s	2.0 × 10^−4^ ± 1.3 × 10^−4^	0.034^1^	
	53	< 10 s	2.0 × 10^−4^ ± 9.0 × 10^−5^		
**At 5.33 s**					
Entropy	41	> 10 s	2.0 × 10^−4^ ± 1.8 × 10^−4^	0.042^1^	
	49	< 10 s	2.0 × 10^−4^ ± 1.2 × 10^−4^		
**At 10.33 s**					
Entropy	36	> 10 s	2.0 × 10^−4^ ± 1.6 × 10^−4^	0.026^1^	
	30	< 10 s	2.0 × 10^−4^ ± 1.0 × 10^−4^		
**LLT**					
**At 0.33 s**					
Total area	28	Grade 1	109.67 ± 8.76	< 0.001^2^	1–3 < 0.001
	38	Grade 2	111.96 ± 8.70		1–4 = 0.002
	15	Grade 3	120.70 ± 10.16		2–3 = 0.001
	13	Grade 4	119.26 ± 7.30		2–4 = 0.008
Minimum pixel intensity	28	Grade 1	60.75 ± 11.06	0.001^2^	1–3 = 0.001
	38	Grade 2	62.60 ± 12.72		1–4 = 0.041
	15	Grade 3	83.79 ± 22.62		2–3 = 0.001
	13	Grade 4	75.92 ± 21.02		2–4 = 0.046
Energy	28	Grade 1	239.86 ± 26.07	< 0.001^2^	1–2 = 0.032 1–3 < 0.001 1–4 ≤ 0.001 2–3 = 0.002 2–4 = 0.001
	38	Grade 2	243.52 ± 22.66		
	15	Grade 3	254.55 ± 2.08		
	13	Grade 4	254.99 ± 0.01		
Relative energy	28	Grade 1	0.33 ± 0.13	< 0.001^2^	1–3 < 0.001
	38	Grade 2	0.40 ± 0.20		1–4 = 0.002
	15	Grade 3	0.73 ± 0.29		2–3 = 0.001
	13	Grade 4	0.72 ± 0.29		2–4 = 0.016
SD irregularity	28	Grade 1	0.03 ± 0.01	< 0.001^2^	1–3 < 0.001
	38	Grade 2	0.05 ± 0.03		1–4 < 0.001
	15	Grade 3	0.18 ± 0.18		2–3 = 0.008
	13	Grade 4	0.15 ± 0.17		2–4 = 0.014
Mean pixel intensity	28	Grade 1	115.09 ± 11.56	< 0.001^2^	1–3 < 0.001
	38	Grade 2	121.47 ± 17.40		1–4 = 0.001
	15	Grade 3	156.54 ± 35.25		2–3 = 0.002
	13	Grade 4	155.23 ± 32.90		2–4 = 0.008
SD of pixel intensity	28	Grade 1	24.50 ± 2.25	< 0.001^2^	1–3 = 0.001
	38	Grade 2	26.06 ± 3.35		1–4 < 0.001
	15	Grade 3	30.37 ± 6.17		2–3 = 0.027
	13	Grade 4	30.84 ± 5.66		2–4 = 0.008
Median pixel intensity	28	Grade 1	113.68 ± 11.76	< 0.001^2^	1–3 < 0.001
	38	Grade 2	119.92 ± 17.47		1–4 = 0.001
	15	Grade 3	156.21 ± 36.16		2–3 = 0.002
	13	Grade 4	157.08 ± 34.84		2–4 = 0.009
Mode pixel intensity	28	Grade 1	120.22 ± 15.53	< 0.001^2^	1–3 < 0.001
	38	Grade 2	122.68 ± 19.12		1–4 = 0.002
	15	Grade 3	178.72 ± 57.03		2–3 < 0.001
	13	Grade 4	178.92 ± 55.12		2–4 = 0.002
Kurtosis	28	Grade 1	0.019 ± 0.003	< 0.001^2^	1–3 < 0.001
	38	Grade 2	0.018 ± 0.003		1–4 < 0.001
	15	Grade 3	0.014 ± 0.003		2–3 = 0.001
	13	Grade 4	0.015 ± 0.003		2–4 = 0.030
Skewness	28	Grade 1	0.15 ± 0.01	< 0.001^2^	1–3 < 0.001
	38	Grade 2	0.15 ± 0.01		1–4 = 0.001
	15	Grade 3	0.13 ± 0.02		2–3 = 0.001
	13	Grade 4	0.13 ± 0.02		2–4 = 0.023
**At 5.33 s**					
Total area	27	Grade 1	110.03 ± 6.85	< 0.001^2^	1–2 = 0.004 1–3 < 0.001 1–4 < 0.001 2–3 = 0.002 2–4 = 0.005
	37	Grade 2	116.70 ± 7.62		
	15	Grade 3	123.84 ± 5.40		
	11	Grade 4	124.25 ± 3.88		
Minimum pixel intensity	27	Grade 1	57.44 ± 8.43	< 0.001^2^	1–2 = 0.001
	37	Grade 2	69.08 ± 14.20		1–3 < 0.001
	15	Grade 3	82.93 ± 19.00		1–4 < 0.001
	11	Grade 4	79.64 ± 14.03		2–3 = 0.023
Energy	27	Grade 1	238.28 ± 23.74	< 0.001^2^	1–2 = 0.007 1–3 < 0.001 1–4 < 0.001 2–3 < 0.001 2–4 < 0.001
	37	Grade 2	242.24 ± 20.14		
	15	Grade 3	254.14 ± 2.28		
	11	Grade 4	254.45 ± 0.39		
Relative energy	27	Grade 1	0.33 ± 0.13	< 0.001^2^	1–2 = 0.005 1–3 < 0.001 1–4 < 0.001 2–3 = 0.002 2–4 = 0.003
	37	Grade 2	0.52 ± 0.23		
	15	Grade 3	0.79 ± 0.20		
	11	Grade 4	0.80 ± 0.17		
SD irregularity	27	Grade 1	0.04 ± 0.01	0.001^2^	1–2 = 0.023 1–3 < 0.001 1–4 < 0.001 2–3 = 0.001 2–4 = 0.001
	37	Grade 2	0.06 ± 0.04		
	15	Grade 3	0.23 ± 0.18		
	11	Grade 4	0.20 ± 0.15		
Mean pixel intensity	27	Grade 1	116.87 ± 9.94	< 0.001^2^	1–2 = 0.005 1–3 < 0.001 1–4 < 0.001 2–3 = 0.002 2–4 = 0.002
	37	Grade 2	132.69 ± 19.70		
	15	Grade 3	166.37 ± 27.89		
	11	Grade 4	166.26 ± 23.58		
SD of pixel intensity	27	Grade 1	27.05 ± 2.86	< 0.001^2^	1–3 < 0.001
	37	Grade 2	28.25 ± 3.58		1–4 < 0.001
	15	Grade 3	34.20 ± 5.77		2–3 = 0.001
	11	Grade 4	34.85 ± 3.59		2–4 < 0.001
Median pixel intensity	27	Grade 1	115.00 ± 10.78	< 0.001^2^	1–2 = 0.004 1–3 < 0.001 1–4 < 0.001 2–3 = 0.001 2–4 = 0.002
	37	Grade 2	131.68 ± 10.78		
	15	Grade 3	166.93 ± 29.20		
	11	Grade 4	166.73 ± 24.05		
Mode pixel intensity	27	Grade 1	116.33 ± 21.15	< 0.001^2^	1–2 = 0.003 1–3 < 0.001 1–4 < 0.001 2–3 = 0.006 2–4 = 0.003
	37	Grade 2	140.35 ± 23.95		
	15	Grade 3	198.64 ± 55.22		
	11	Grade 4	202.90 ± 52.29		
Kurtosis	27	Grade 1	0.017 ± 0.001	< 0.001^2^	1–3 < 0.001
	37	Grade 2	0.016 ± 0.002		1–4 < 0.001
	15	Grade 3	0.012 ± 0.002		2–3 < 0.001
	11	Grade 4	0.012 ± 0.002		2–4 = 0.001
Skewness	27	Grade 1	0.14 ± 0.01	< 0.001^2^	1–3 < 0.001
	37	Grade 2	0.14 ± 0.01		1–4 < 0.001
	15	Grade 3	0.12 ± 0.01		2–3 < 0.001
	11	Grade 4	0.12 ± 0.01		2–4 = 0.001
**At 10.33 s**					
Total area	18	Grade 1	110.14 ± 8.54	< 0.001^2^	1–3 < 0.001
	28	Grade 2	115.47 ± 8.34		1–4 < 0.001
	11	Grade 3	125.88 ± 3.45		2–3 < 0.001
	9	Grade 4	124.59 ± 2.56		2–4 = 0.012
Minimum pixel intensity	18	Grade 1	58.00 ± 11.69	< 0.001^2^	1–3 < 0.001 1–4 = 0.004 2–3 = 0.001
	28	Grade 2	66.43 ± 15.16		
	11	Grade 3	91.64 ± 19.27		
	9	Grade 4	88.33 ± 14.93		
Energy	18	Grade 1	238.22 ± 20.54	< 0.001^2^	1–3 < 0.001
	28	Grade 2	242.01 ± 19.16		1–4 < 0.001
	11	Grade 3	254.01 ± 2.09		2–3 = 0.001
	9	Grade 4	254.04 ± 0.42		2–4 = 0.004
Relative energy	18	Grade 1	0.36 ± 0.18	< 0.001^2^	1–3 < 0.001
	28	Grade 2	0.50 ± 0.25		1–4 < 0.001
	11	Grade 3	0.89 ± 0.16		2–3 < 0.001
	9	Grade 4	0.80 ± 0.13		2–4 = 0.010
SD irregularity	18	Grade 1	0.04 ± 0.01	< 0.001^2^	1–3 < 0.001
	28	Grade 2	0.07 ± 0.05		1–4 < 0.001
	11	Grade 3	0.29 ± 0.21		2–3 < 0.001
	9	Grade 4	0.21 ± 0.13		2–4 = 0.004
Mean pixel intensity	18	Grade 1	117.99 ± 13.74	< 0.001^2^	1–3 < 0.001
	28	Grade 2	132.00 ± 22.31		1–4 < 0.001
	11	Grade 3	178.50 ± 25.59		2–3 < 0.001
	9	Grade 4	170.99 ± 19.92		2–4 = 0.003
SD of pixel intensity	18	Grade 1	27.04 ± 2.96	< 0.001^2^	1–3 < 0.001
	28	Grade 2	29.48 ± 3.57		1–4 < 0.001
	11	Grade 3	33.99 ± 4.04		2–3 = 0.006
	9	Grade 4	35.10 ± 4.34		2–4 = 0.004
Median pixel intensity	18	Grade 1	116.17 ± 14.40	< 0.001^2^	1–3 < 0.001
	28	Grade 2	130.82 ± 23.06		1–4 < 0.001
	11	Grade 3	179.45 ± 27.88		2–3 < 0.001
	9	Grade 4	170.67 ± 20.63		2–4 = 0.004
Mode pixel intensity	18	Grade 1	117.67 ± 23.25	< 0.001^2^	1–3 < 0.001
	28	Grade 2	137.79 ± 31.60		1–4 < 0.001
	11	Grade 3	214.36 ± 49.28		2–3 < 0.001
	9	Grade 4	192.67 ± 49.86		2–4 = 0.002
Kurtosis	18	Grade 1	0.017 ± 0.002	< 0.001^2^	1–3 < 0.001
	28	Grade 2	0.015 ± 0.003		1–4 = 0.001
	11	Grade 3	0.012 ± 0.002		2–3 = 0.001
	9	Grade 4	0.013 ± 0.003		2–4 = 0.015
Skewness	18	Grade 1	0.14 ± 0.01	< 0.001^2^	1–3 < 0.001
	28	Grade 2	0.13 ± 0.01		1–4 = 0.001
	11	Grade 3	0.11 ± 0.01		2–3 = 0.001
	9	Grade 4	0.11 ± 0.01		2–4 = 0.011

*LLT* lipid layer thickness, *m* millimetres, *n* number of patients, *NIKBUT* non-invasive keratograph break-up time, *s* seconds, *SD* standard deviation, *TMH* tear meniscus height

^1^Mann-Whitney *U* test

^2^Kruskal-Wallis test

### Multiple linear regressions

Since the metrics at 5.33 s after blinking have proved to differentiate between grades 1 (open meshwork), 2 (closed meshwork) and 3 (wave) of the LLT, only the metrics at 5.33 s after blinking will be assessed in this section of the manuscript.

Multiple linear regressions (Table 5) were performed to show the current metrics that were associated with new metrics, avoiding that the interaction between current metrics mislead results. Multiple linear regressions showed that new metrics were statistically significant associated with LLT, explaining the variability between 7.1 and 47.0% depending on the metric. Kurtosis and skewness showed a weak association with gland drop-out percentage instead of with LLT. Energy also appeared to be associated with the first median NIKBUT together with LLT. No association was found with the remaining variables. Generally, these results suggest that the main predictor factor of new metrics was LLT.

**Table 5:** Multiple linear regressions for new metrics at 5.33 s where the independent variables included were gland drop-out percentage, bulbar redness, lipid layer thickness, tear meniscus height, first and mean NIKBUT, gland expressibility, OSDI and DEQ-5

New metrics	Current metrics	*β*	SE	Sβ	Significance level	Adjusted *R* square
Total area	Constant	129.99	140.09		< 0.001	0.470
	LLT	18.64	2.62	0.71	< 0.001	
Minimum pixel intensity	Constant	40.34	9.86		< 0.001	0.325
	LLT	9.72	1.83	0.60	< 0.001	
Energy	Constant	261.47	8.09		< 0.001	0.214
	LLT	5.27	1.50	0.42	0.001	
	First median NIKBUT	1.24	0.61	0.64	0.045	
Relative energy	Constant	0.87	0.14		< 0.001	0.404
	LLT	0.17	0.03	0.69	< 0.001	
Entropy	Constant	0.000	0.000		< 0.001	0.050
	First median NIKBUT	0.00000063	0.00	0.73	0.037	
SD irregularity	Constant	0.13	0.03		< 0.001	0.071
	LLT	0.014	0.005	0.39	0.005	
Mean pixel intensity	Constant	137.78	15.63		< 0.001	0.457
	LLT	20.26	2.91	0.70	< 0.001	
SD of pixel intensity	Constant	25.15	1.81		< 0.001	0.193
	LLT	1.22	0.34	0.45	0.001	
Median pixel intensity	Constant	140.60	16.64		< 0.001	0.468
	LLT	21.89	3.09	0.70	< 0.001	
Mode pixel intensity	Constant	130.92	22.42		< 0.001	0.432
	LLT	28.37	4.17	0.70	< 0.001	
Kurtosis	Constant	0.012	0.001		< 0.001	0.114
	Gland drop-out percentage	0.000031	0.000	0.33	0.042	
Skewness	Constant	0.119	0.005		< 0.001	0.099
	Gland drop-out percentage	0.000	0.000	0.36	0.029	

*β* unstandardized coefficient, *Sβ* standardized coefficient, *LLT* lipid layer thickness, *NIKBUT* non-invasive break-up time, *SD* standard deviation, *SE* standard error

### Diagnostic capability and validation of the new metrics

Table 6 summarizes the diagnostic power and the cut-off values for each new metric when grade 1 LLT was compared with other grades. New developed metrics were powerful indicators to detect subjects with an altered lipid layer (grade 1 — open meshwork) since the area under the curve, sensitivity and specificity obtained were high. Mean pixel intensity, median pixel intensity and relative energy were the metrics with the highest sensitivity, specificity, area under the curve, Youden index, discriminant power, accuracy, Kappa index and *F*-measure.

**Table 6:** ROC curve parameters of newly developed metrics to differentiate grade 1 LLT from other grades at 5.33 s

Metric	Sensitivity	Specificity	Area under the curve (CI)	Cut-off value	Youden index	Discriminant power	Accuracy	Kappa index	*F*-measure
Total area	0.94	0.76	0.89 (0.83–0.96)	117.74	0.70	2.18	0.83	0.72	0.81
Minimum pixel intensity	0.92	0.74	0.88 (0.82–0.95)	67.50	0.65	1.89	0.82	0.70	0.80
Energy	0.87	0.77	0.82 (0.71–0.88)	239.15	0.65	1.77	0.82	0.70	0.80
Relative energy	0.92	0.81	0.91 (0.83–0.96)	0.48	0.73	2.13	0.86	0.76	0.84
SD irregularity	0.89	0.77	0.86 (0.79–0.94)	0.05	0.66	1.83	0.82	0.70	0.79
Mean pixel intensity	0.94	0.79	0.89 (0.83–0.96)	128.62	0.74	2.29	0.86	0.76	0.84
SD of pixel intensity	0.83	0.70	0.78 (0.68–0.88)	28.08	0.53	1.35	0.74	0.57	0.73
Median pixel intensity	0.92	0.81	0.91 (0.84–0.97)	124.50	0.73	2.13	0.86	0.76	0.84
Mode pixel intensity	0.83	0.77	0.87 (0.80–0.94)	133.50	0.61	1.56	0.80	0.66	0.78
Kurtosis	0.89	0.76	0.83 (0.74–0.92)	0.015	0.64	1.77	0.78	0.63	0.76
Skewness	0.92	0.72	0.84 (0.75–0.92)	0.13	0.63	1.84	0.80	0.66	0.78

*CI* 95% confidence interval, *SD* standard deviation

Tables 7 and 8 show the diagnostic power of each new metric to differentiate between grades 1 and 2, and between grades 2 and 3, respectively. This step allowed finding the cut-off values for each new metric to objectively classify the lipid layer into different grades. The cut-off value which optimizes the diagnosis determines the best score to diagnose the disease. Thus, a subject with a higher score than the cut-off value in kurtosis and skewness was classified into the thinner LLT group, while a subject with a higher score than the cut-off value in the rest of the newly developed metrics was classified into the thicker LLT group. The SD of pixel intensity had a low specificity to distinguish between grades 1 and 2, which could lead to the lipid layer being misclassified.

**Table 7:** ROC curve parameters of new developed metrics to differentiate between grades 1 and 2 LLT at 5.33 s

Metric	Sensitivity	Specificity	Area under the curve (CI)	Cut-off value	Youden index	Discriminant power
Total area	0.89	0.70	0.83 (0.73–0.94)	116.20	0.59	1.62
Minimum pixel intensity	0.86	0.74	0.86 (0.77–0.95)	64.50	0.60	1.58
Energy	0.86	0.73	0.81 (0.71–0.91)	238.59	0.59	1.55
Relative energy	0.92	0.74	0.83 (0.72–0.94)	0.48	0.66	1.90
SD irregularity	0.83	0.70	0.78 (0.66–0.90)	0.05	0.54	1.36
Mean pixel intensity	0.92	0.74	0.84 (0.73–0.95)	126.93	0.66	1.90
SD of pixel intensity	0.83	0.48	0.66 (0.53–0.80)	28.22	0.31	0.84
Median pixel intensity	0.92	0.74	0.85 (0.74–0.95)	124.50	0.66	1.90
Mode pixel intensity	0.83	0.70	0.82 (0.71–0.93)	133.50	0.54	1.36
Kurtosis	0.89	0.63	0.74 (0.60–0.87)	0.015	0.52	1.44
Skewness	0.83	0.67	0.74 (0.60–0.87)	0.14	0.5	1.27

*CI* 95% confidence interval, *SD* standard deviation

**Table 8:** ROC curve parameters of new developed metrics to differentiate between grade 2 and 3 LLT at 5.33 s

Metric	Sensitivity	Specificity	Area under the curve (CI)	Cut-off value	Youden index	Discriminant power
Total area	0.78	0.81	0.80 (0.65–0.95)	123.97	0.59	1.50
Minimum pixel intensity	0.71	0.69	0.74 (0.57–0.91)	79.50	0.40	0.94
Energy	0.71	0.84	0.78 (0.62–0.93)	248.19	0.55	1.41
Relative energy	0.74	0.88	0.81 (0.67–0.96)	0.72	0.62	1.65
SD irregularity	0.89	0.69	0.82 (0.68–0.97)	0.12	0.58	1.58
Mean pixel intensity	0.78	0.88	0.83 (0.68–0.97)	150.69	0.65	1.76
SD of pixel intensity	0.82	0.69	0.80 (0.65–0.95)	32.36	0.50	1.25
Median pixel intensity	0.70	0.88	0.83 (0.69–0.97)	160.50	0.58	1.55
Mode pixels	0.96	0.63	0.80 (0.63–0.96)	183.00	0.59	2.08
Kurtosis	0.82	0.75	0.82 (0.68–0.96)	0.013	0.57	1.42
Skewness	0.93	0.69	0.84 (0.70–0.97)	0.12	0.61	1.83

*CI* 95% confidence interval, *SD* standard deviation

Once the cut-off values were calculated, the lipid layer was objectively classified. The level of agreement between the newly developed objective and existing subjective classifications was evaluated (Table 9). Since different LLT grades were evaluated, the weighted Kappa index was calculated [37]. Mean pixel intensity, median pixel intensity and relative energy were the metrics with the highest area under the curve, best relationship between sensitivity and specificity and higher agreement between objective and subjective methods for LLT classification.

**Table 9:** Agreement between the subjective and objective classification of LLT for each parameter at 5.33 s

Metric	Accuracy	Kappa index	*F*-measure
Total area	0.72	0.73	0.84
Minimum pixel intensity	0.68	0.66	0.81
Energy	0.72	0.73	0.84
Relative energy	0.76	0.76	0.86
SD irregularity	0.71	0.73	0.83
Mean pixel intensity	0.77	0.77	0.87
SD of pixel intensity	0.63	0.61	0.78
Median pixel intensity	0.76	0.77	0.86
Mode pixel intensity	0.71	0.67	0.83
Kurtosis	0.69	0.69	0.82
Skewness	0.70	0.69	0.82

*SD* standard deviation

## Discussion

The assessment of LLT plays an essential role in DED and MGD because of the relevance of the lipid layer in the TF [1, 4]. Existing tests lack objectivity, preciseness, are time-consuming or are inaccessible for most clinicians due to the need of an interferometer to be performed [8, 10, 11, 13–19]. The present article introduces a new self-developed technique for the non-invasive objective evaluation of the LLT which can be implemented in any Placido disk topograph.

The present work has tested the validity and applicability of new metrics calculated from the grey level intensity values of the Placido disk pattern reflected onto the TF. Alonso-Caneiro et al. [22] performed a similar study, in which they used texture analysis of videokeratoscopy images and denoted that the proposed technique offered clinical utility in the diagnosis of DED (area under the curve from 0.77 to 0.82, sensitivity of 0.9 and specificity of 0.6). However, the authors did not explain why this could be a predictor of DED since they did not study the correlations of the metric with ocular surface parameters. Therefore, they did not evidence which parameter of the TF they were measuring.

The present work makes three important contributions: (1) the development of a new method to assess LLT in an unbiased, objective, quick and non-invasive way; (2) the possibility of assessing the lipid layer without the need of an interferometer, making the method widely accessible; (3) the validation of the new technique through the study of its repeatability, diagnostic capability and correlations with ocular surface parameters.

### Correlations between Placido disk reflectivity metrics and ocular surface parameters

Moderate positive significant correlations were found between grey level intensities of the Placido disk pattern and LLT and NIKBUT. The correlations between new developed metrics and age, meibomian glands drop-out, bulbar redness, TMH and OSDI (Table 3) might be a consequence of their correlation with LLT since LLT is also correlated with age, meibomian glands drop-out and NIKBUT [38–42].

Despite the above, in the present study, LLT revealed no correlation with bulbar redness, TMH and OSDI. Finis et al.[41] neither found a significant correlation between DED symptoms and LLT, although this was not in accordance with others [39, 40, 43, 44]. New metrics, though less strongly correlated with bulbar redness, TMH and OSDI than with LLT, could still be used to assess these ocular surface parameters.

Entropy measures the randomness of a grey level distribution [22] and as a result might change as the TF becomes thinner and the Placido disk pattern becomes more unstructured [22]. This metric was not correlated with LLT, although it revealed a significant correlation with glands drop-out, bulbar redness, TMH, NIKBUT and OSDI, and thus, it might be used to predict these parameters.

Moreover, despite that new metrics were correlated with LLT, no statistically significant correlations were found with meibomian glands expressibility, although previous research did find a correlation between these parameters [41].

### Differences between groups

When the sample was subjectively divided into 4 different LLT groups, using grade scales of interference patterns, statistically significant differences in the new metrics were found between them (Table 4). The measurements at 5.33 s after blinking were the best to differentiate among the different LLT grades since metrics were able to distinguish between grades 1 and 2 and grades 2 and 3. Nonetheless, the algorithm could not differentiate between grades 3 (wave) and 4 (colour fringe pattern). This could be due to the fact that grade 4 differs from grade 3 in that 4 is the only grade, in the interference scale, to imply a coloured pattern, which cannot be detected using grey level values. Hence, as already reported by other authors [8], it would be necessary to incorporate a colour analysis to differentiate between grades 3 and 4.

Nevertheless, since the TFOS DEWS II diagnostic report reported that a subject is classified as having DED when the LLT has a grade of 1, differentiating between grade 3 and 4 has a low clinical utility. Additionally, thinner patterns are more difficult to characterize by an examiner [3, 14].

In addition to being capable of differentiating between LLT grades, the metrics at 5.33 s after blinking are performed under more realistic conditions than at later times, as subjects are not required to forcefully suppress blinking. Moreover, metrics at 0.33 s might not have achieved a similar performance than at 5.33 s in assessing LLT since at 0.33 s after blinking, the lipid layer might not have stabilized yet.

### Placido disk reflectivity metrics over time

Repeated mixed model ANOVA showed statistical higher pixel intensity values at 10.33, 15.33 and 20.33 s than at 0.33 s (Table 1). This might be due to the fact that the sample size decreased as the seconds after blink increased. Thus, only subjects with larger NIKBUT values were able to maintain the eye opened for 20.33 s. This may be behind the observed differences as LLT and NIKBUT were positively correlated with pixel intensity.

Nevertheless, despite that ANOVA revealed differences in the metrics between periods, when all subjects were analysed together, CoV, which evaluated the variability in each subject individually, revealed a low variability of metrics over time.

### Repeatability of each Placido disk reflectivity metric

The present method has the limitation that is semiautomatic since the centre of the Placido disk pattern and the ROI must be selected manually by the examiner. In spite of this, the repeatability was acceptable in all metrics (Table 2) and the analysis can be carried out in less than 10 s. It has been previously reported that this time is considered appropriate for a clinical test [45].

### Multiple linear regressions

As correlations showed, LLT was the clinical parameter that was more strongly correlated with new metrics. Nevertheless, other parameters were also correlated. This could be a bias since different metrics can confound results, affecting the classification of LLT. Therefore, multiple linear regression analysis has been performed to show which current metrics are independently associated with new metrics. Results showed that for most metrics, LLT was the only parameter associated. This suggests that new metrics are predictors of LLT and can be used to objectively assess it. Nevertheless, kurtosis and skewness were associated with gland drop-out and energy with LLT together with NIKBUT.

### Diagnostic capability and validation of the new metrics

ROC curves were calculated to analyse the diagnostic ability of the new metrics. It has been previously reported that a 70% level of sensitivity and specificity is acceptable for the diagnosis of a disease [6]. Sensitivity and specificity were higher than 0.7 for most of the developed new metrics.

According to the classification on previous reports [46], the newly developed metrics showed areas under the curve between acceptable (0.74) and outstanding (0.91) discrimination. Thus, new metrics can be considered powerful aides to objectively assess the lipid layer.

It has been reported that accuracy, *F*-measure and kappa index denote good agreement between tests when they are close to 1 [33–37]. Generally, the agreement between new metrics and subjective classification methods of LLT showed an accuracy between 0.63 and 0.77, an *F*-measure between 0.78 and 0.87 and a Kappa index between 0.61 and 0.77 (very good agreement) (Table 8).

Mean pixel intensity, median pixel intensity and relative energy at 5.33 s after blinking were the metrics with the highest diagnostic capability in terms of sensitivity, specificity, area under the curve, Youden index and discriminant power (Table 5) and the metrics with the highest agreement with the subjective grading in terms of accuracy, Kappa index and *F*-measure (Table 8).

In comparison with previous studies on the analysis of interference patterns [8, 10–18], the new metrics showed slightly lower diagnostic ability and agreement with the subjective classification of LLT. Nevertheless, this method adds the possibility of objectively assessing the LLT without the need of having an interferometer, which might broad the assessment of the lipid layer in clinical practice.

This study had some limitations to consider. First, statistically significant correlations between new metrics and age were found. Consequently, age might act as a possible confounding factor. As in previous studies, age could not be excluded from the analysis because of its strong association with DED and MGD [39, 47]. Furthermore, the surrounding illumination and the focussing of the Placido disk pattern should be carefully controlled. In addition, LLT has not been measured objectively. However, it has been measured subjectively with a validated grading scale, which suggests that the present method is able to objectify the subjective measurement of this grading scale. It has been reported that this subjective grading scale is correlated with LLT [3, 4, 6, 7]. Therefore, these issues are not expected to affect results significantly. Future studies could assess the predictability of LLT measured objectively with the new metrics. Finally, the method only measures the grey intensity values of the Placido disk pattern within the pupil. Nevertheless, this issue is not expected to influence the outcomes since all the metrics have been designed to be pupil-independent. Moreover, the present study has demonstrated that the analysis of the pixels within the pupil area is enough to assess LLT.

## Conclusions

Overall, the analysis of grey level intensity values in videokeratography is able to assess TF behaviour. Grey level intensity can be used as an alternative biomarker to objectively grade LLT. It has been demonstrated that the method is quick, objective, non-invasive, repeatable and with acceptable sensitivity and specificity. Therefore, it could be easily included in a battery of tests to improve the detection and monitoring of DED and MGD in clinical practice.

Further research is needed to assess the performance of these metrics in subjects diagnosed with DED or MGD. Likewise, the software could be further developed to be fully automatic and to distinguish between grades 3 and 4 of LLT. Nonetheless, although these outcomes are preliminary, they are highly encouraging. This study could be the base for future works which attempt to assess LLT objectively without the need of an interferometer.

## Abbreviations

CI: Confidence interval
CoR: Coefficient of repeatability
CoV: Coefficient of variation
DED: Dry eye disease
DEQ-5: Dry Eye Questionnaire-5
LLT: Lipid layer thickness
MGD: Meibomian gland dysfunction
NIKBUT: Non-invasive keratograph break-up time
OSDI: Ocular Surface Disease Index
ROC: Receiver operating characteristics
ROI: Region of interest
SD: Standard deviation
*S*_w_: Within-subject standard deviation
TF: Tear film
TFOS DEWS II: Tear Film and Ocular Surface Dry Eye Workshop II
TMH: Tear meniscus height
